# Use of ELISpot assay to study HBs-specific B cell responses in vaccinated and HBV infected humans

**DOI:** 10.1038/s41426-018-0034-0

**Published:** 2018-02-16

**Authors:** Chen Tian, Yuxin Chen, Yong Liu, Shixia Wang, Yang Li, Guiyang Wang, Juan Xia, Xiang-an Zhao, Rui Huang, Shan Lu, Chao Wu

**Affiliations:** 1 Department of Infectious Diseases, Nanjing Drum Tower Hospital, Medical School of Nanjing University, Nanjing, Jiangsu 210008 China; 2 Department of Laboratory Medicine, Nanjing Drum Tower Hospital, Medical School of Nanjing University, Nanjing, Jiangsu 210008 China; 3 Department of Experimental Medicine, Nanjing Drum Tower Hospital, Medical School of Nanjing University, Nanjing, Jiangsu 210008 China; 4 Department of Medicine, University of Massachusetts Medical School, Worcester, MA 01605 USA

## Abstract

Hepatitis B surface antibody (HBsAb) plays a critical role in protecting against infection of hepatitis B virus (HBV) and were extensively studied in literature. At the same time, the status of hepatitis B surface antigen (HBs)-specific B cells in both vaccinated and HBV infected people received limited attention. In the current study, we adopted a highly specific B-cell Enzyme Linked ImmunoSpot (ELISpot) assay to analyze HBs-specific B cells in various clinical settings: healthy individuals with the history of HBV vaccination before and after receiving an extra HBV vaccine boost, people chronically infected with HBV (CHB) in various clinical stages, with or without a particular type anti-viral treatment, or whether receiving a dose of HBV vaccine. In all of these cases, B-cell ELISpot assay was used effectively in enumerating the frequency of HBs-specific B cells. While the focus of the current report was to establish the utility of this assay for HBV research, a number of interesting observations were made in this pilot study based on the profiles and dynamics of HBs-specific B cells in various conditions. Such information is useful to guide the future work in designing novel therapeutic strategies against CHB.

## Introduction

Hepatitis B virus (HBV) infection remains a major health threat in many parts of the world especially in developing countries including countries with large human populations such as China^1^. While the introduction of a subunit protein-based HBV vaccine has greatly reduced the rate of new human infections in the last several decades, the population of people who either were infected before the introduction of HBV vaccine or missed the chance of getting vaccinated is still quite large^2^. Existing therapies can only partially control the infection but not cure the diseases^3,4^. Finding novel treatment strategies especially an immune therapy is critically needed.

The significance of vaccine-induced antibody (HBsAb) responses against HBV surface antigen (HBsAg) in protecting against HBV infection has been well established^5,6^. However, HBsAb is basically missing in chronic HBV-infected patients. How to break body’s immune tolerance to elicit protective HBsAb that can control the viral infection is a major challenge in HBV research.

The level of serum HBsAb has been used as the gold standard in determining the success of HBV vaccination^7^. Scientists have also assigned the induction of serum HBsAb as the biomarker for clinical cure of HBV infection. However, since most experimental immune therapies have not achieved this goal, there is a great need to develop alternative biomarkers to detect any early signs of immune activation against HBV infection. In theory, HBsAb is produced by hepatitis B surface antigen (HBs)-specific B cells and the presence of HBs-specific memory B cells may be an indicator of potential HBsAb responses. Unfortunately, such tests have not been well established or widely used to monitor the level of circulating HBs-specific memory B cells in human peripheral blood.

In the current study, we investigated the levels of HBs-specific B cells in the peripheral blood of 21 HBV vaccine immunized healthy individuals and 67 patients with different immunological phases of chronic HBV infection. The relationship between the titer of serum HBsAb and the level of HBs-specific B cells was analyzed. Furthermore, the dynamic change of HBs-specific memory B cells after either vaccination or antiviral treatment was monitored in this pilot study. Information learned from the current study would be useful to better understand the basic immunological mechanisms that are involved in the induction and maintenance of HBsAb as part of the effort to develop novel immune therapies against chronic HBV infection.

## Results

### HBs-specific memory B cells in healthy adults with history of HBV vaccination

**Fig. 1:**
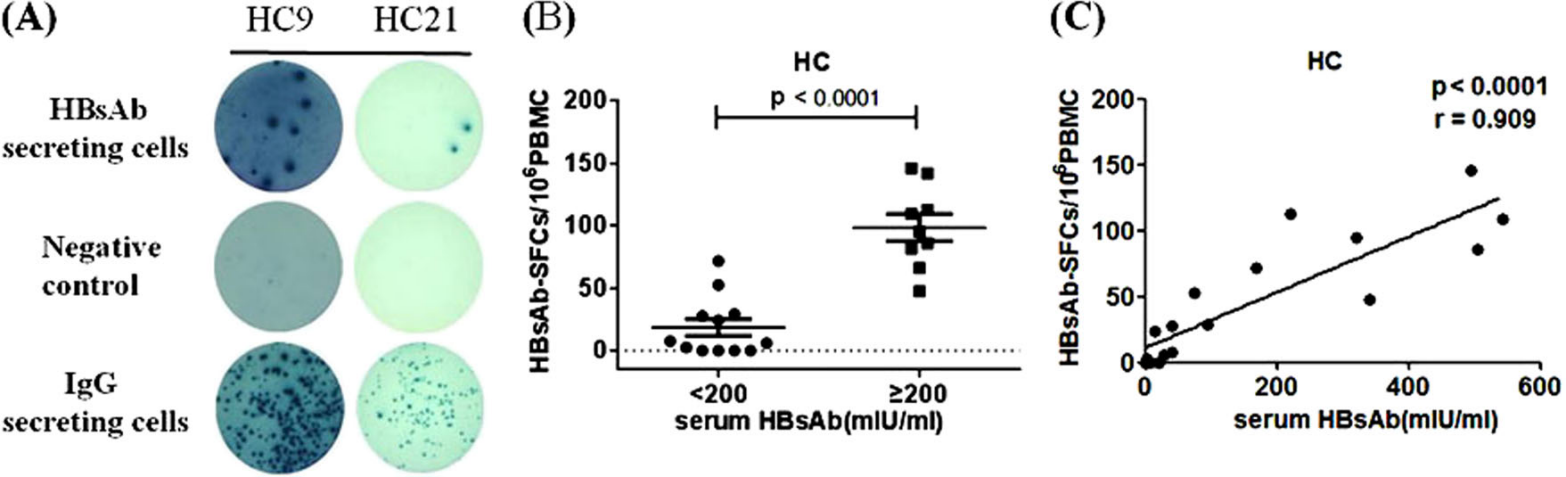
HBs-specific B cells in health volunteers. **a** Representative B-cell ELISpot readouts from healthy volunteers HC9 and HC21. 100,000 cells were added in HBsAg-coated wells (up panel) and negative wells (middle panel) and 5000 cells were added in IgG coated wells (bottom panel). **b** The frequency of HBs-specific memory B cells was significantly reduced in groups with lower level of serum HBsAb (<200 mIU/ml), compared to the groups with higher serum HBsAb ( > 200 mIU/ml). **c** A strong correlation between the serological HBsAb and the number of memory B cell was detected in HCs. SFC, spot-forming cells. Each dot represents one individual

A sensitive B-cell ELISpot assay was used in the current study to detect and enumerate HBs-specific memory B cells from human peripheral blood mononuclear cells (PBMCs) among healthy adult vaccinees. Human PBMCs were cultured for 5 days in the presence of R848 and IL-2. The resting memory B cells start secreting detectable level of specific antibodies after receiving ex vivo polyclonal activation^8^. Among people who had been vaccinated with HBV vaccine in the past, HBs-specific memory B cells would be detected by the HBs-specific B cell ELISpot assay. HBsAb secreted by these B cells were captured by recombinant HBsAg antigen coated on the ELISpot plates, further demonstrated by colored spots revealed on the plates following the addition of biotinylated anti-human IgG antibody and HRP-conjugated streptavidin. As shown in Fig. 1a, representative B cell ELISpot assay results revealed HBsAb-secreting B cells from two individuals (HC9 and HC21) in the healthy control (HC) group. The frequency of HBsAb-secreting B cells in HC9 was higher than that in HC21, reflecting the variation of HBs-specific memory B cells in vaccinees. The positive control wells using PBMCs from the same two individuals showed much higher frequency of non-specific total IgG-secreting B cells when anti-human IgG was used as the capture reagent. Wells without any capturing reagent were used as the negative controls (Fig. 1a).

Among 21 individuals included in the HC group, 17 of them (80.95%) had positive HBsAb-secreting B cells, despite they all had reported history of HBV vaccination at least 2 years ago. This result fit the reports that about 20% of vaccinated people were non-responders^9–11^. The value of HBsAb-secreting B-cell measurement was further confirmed based on the association between serological HBsAb level and frequency of HBsAb-secreting B cells. Vaccinees with >200 mIU/mL of serum HBsAb had a significantly higher level of HBsAb-secreting B cells compared to those with <200 mIU/mL of serum HBsAb (98.33 ± 10.89 vs.18.4 ± 6.81 per 10^6^ PBMCs; *p* < 0.0001; Fig. 1b). A strong correlation was observed between HBsAb level and the frequency of HBsAb-secreting B cells among vaccinees (*p* < 0.0001, *r* = 0.909; Fig. 1c). In the latter analysis, only data from 15 healthy individuals were included because the rest of people had serum HBsAb level which was too high to be quantified by the particular measurement process used in the study. Therefore, at least within the optimal measurement range, the level of blood HBsAb was proportional to the frequency of circulating HBs-specific memory B cells among healthy vaccinated people.

### Dynamic change of HBs-specific B cells following boosting vaccination

**Fig. 2:**
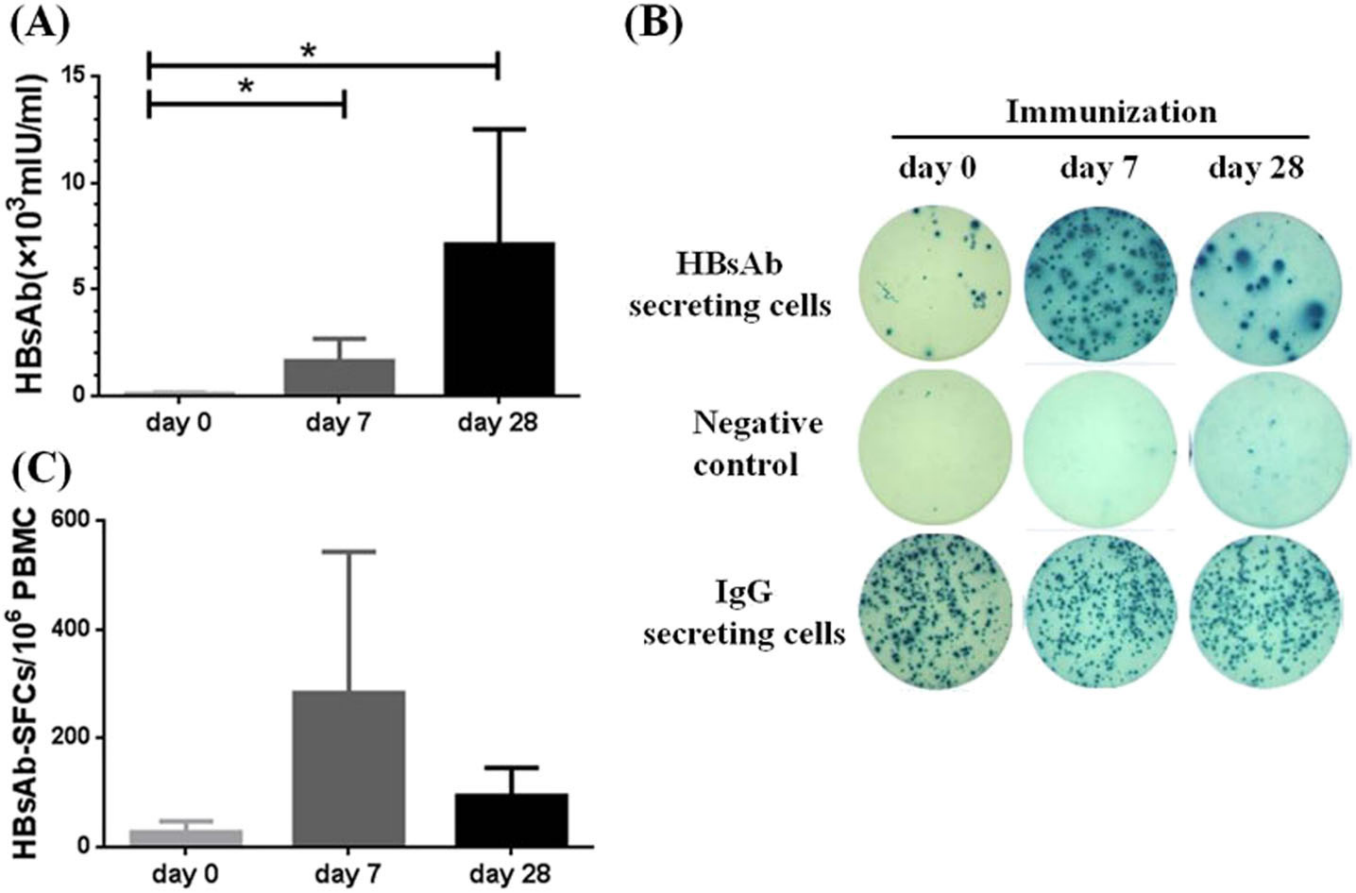
HBs-specific B cells in healthy volunteers post immunization. **a** Serum HBsAb titers increased persistently in all immunized healthy subjects at day 7 and day 28 post HBV vaccine boost. **b** Representative B-cell ELISpot readouts (HC18), who had already finished HBV vaccine immunization 2 years ago, at day 0 (top), day 7 (middle) and day 28 (bottom) after HBV vaccine boost were shown. **c** HBs-specific B-cell responses were measured either at day 7 or day 28 after boost. *P < 0.05

To further evaluate whether HBs-specific B-cell ELISpot assay can be used to monitor the short-term immune response after vaccination, 7 out of 21 healthy individuals who received three doses of HBV vaccines during their childhood but had low level HBsAb responses in our study agreed to receive one additional HBV vaccine boost. Both the titer of HBsAb responses and the frequency of HBsAb-secreting B cells were measured on day 0 (prior to immunization) and at days 7 and 28 post the HBV vaccine boost. As expected, all subjects had substantial increase in serum HBsAb titers at day 7 post vaccine boost and HBsAb titers went even higher at day 28 post boost (Fig. 2a). As illustrated by one representative B-cell ELISpot result from the healthy volunteer HC18, a pronounced elevation of HBsAb-secreting B cells was observed at day 7 post the boost (Fig. 2b), which is mainly contributed by the response of plasmablast B-cell population typical with the peak time around 7 days after the boosting immunization in a previously vaccinated host^12^. The HBsAb-secreting B-cell levels came down quite quickly to a lower level at day 28 post the boost but remained elevated, which represent the further expansion of HBs-specific memory B-cell population (Fig. 2b). While the elevation of HBsAb-secreting B cells varied somewhat among these seven individuals, the group average demonstrated the same pattern of a quick elevation at day 7 and maintained at a lower but still elevated level at day 28 post boost (Fig. 2c).

The above results confirmed that a HBV vaccine could elevate the HBsAb secreting B cells with pronounced peak at day 7, and boost antibody titers with a later peak time. In a previously immunized healthy population, each boost immunization should expand both plasmablast/plasma cells and the memory B cells. Plasmablast/plasma cells are directly responsible for the quick production of antibodies. Increase of memory B cells on the other hand will expand the pool of HBs-specific IgG-secreting B cells for later even more pronounced antibody responses. In our study, B-cell ELISpot assay is accurate and sensitive to quantitatively demonstrate the dynamic changes of HBs-specific B cells in healthy individuals.

### Detection of HBs-specific B cells in chronic HBV-infected patients

**Fig. 3:**
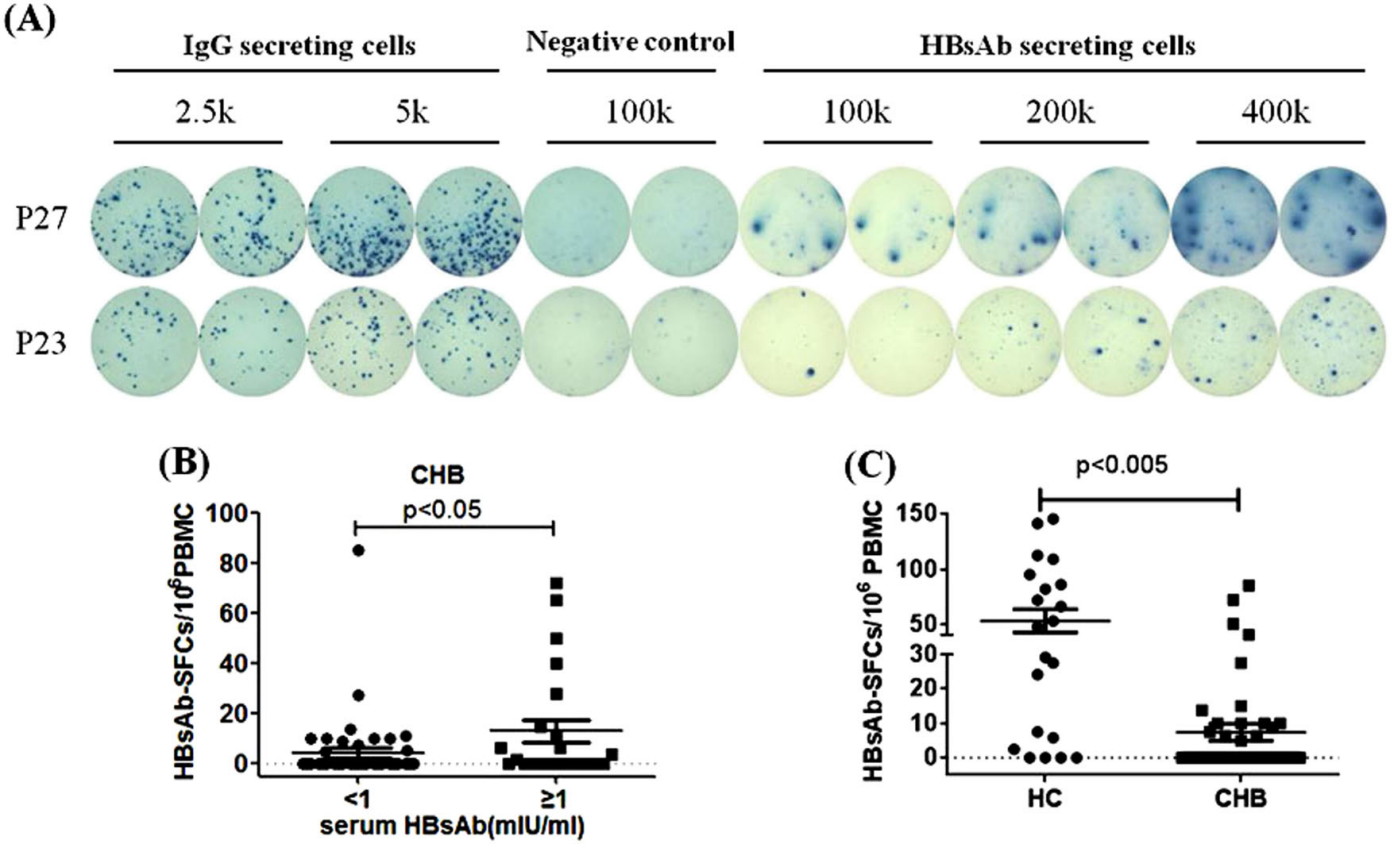
HBs-specific B cells in chronic hepatitis B (CHB) patients. **a** RepresentativeELISpot data from two CHB patients P27 and P23 were shown. Each condition was performed in duplicates. **b** Patients with lower level of serum HBsAb (<1 mIU/ml) had relatively fewer HBsAg-specific B cells, compared to those patients with higher level of serum HBsAb (>1 mIU/mL). **c** The numbers of HBs-specific B cells among 10^6^ PBMC population were shown in each individual from HC group and CHB patient group

We next tried to determine whether our assay was sensitive to detect any HBs-specific B cells present in the peripheral blood of chronic hepatitis B infected (CHB) patients. To our surprise, HBsAb-secreting B cells could be easily detected in certain CHB patients as shown by the representative data from two CHB patients in Fig. 3a. In our study, 16 (31.4%) out of 51 CHB patients had positive HBsAb-secreting B cells as revealed by B-cell ELISpot assay. Further investigation found that some of these patients also had detectable HBsAb in their sera, and there was a general relationship between the serum level of HBsAb and the frequency of HBs-specific B cells in CHB patients. CHB patients with relatively high level of HBsAb (>1mIU/mL) had significantly elevated level of HBs-specific B cells than CHB patients with low HBsAb level (<1mIU/mL) (14.58 ± 6.61 vs.4.69 ± 2.24 per 10^6^ PBMCs) (*p* < 0.05) (Fig. 3b). However, we did not find a linear correlation between serum HBsAb and the frequency of HBs-specific B cells among CHB patients (*p* = 0.811, r = 0.135) (data not shown). This finding may reflect different B cell dynamics in CHB patients who receive constant re-stimulation by the viral antigens. More significantly, the frequency of HBsAb-secreting B cells was significantly lower in CHB patients than in healthy vaccinated individuals (7.4 ± 2.5 vs 52.66 ± 10.64 per 10^6^ PBMCs) (*p* < 0.0001, Fig. 3c).

Once we observed the presence of HBs-specific B cells in the peripheral blood of CHB patients, we next studied whether such cells were only present in certain subgroups of HBV patients during their natural history of infection. According to 2012 EASL guidelines^13^, the three major phases among CHB infection are immune tolerant phase (IT), immune reactive HBeAg-positive phase (IA) and inactive HBV carrier state (IC). IT phase is characterized by a high level of HBV replication whereas there is no or only mild liver necroinflammation. IA phase has relatively low level of replication compared to IT stage, but has an increased or fluctuating level of aminotransferase, moderate or severe liver necroinflammation and more rapid progression of fibrosis. IC phase is characterized by low or undetectable serum HBV DNA along with normal serum aminotransferase. In our study a total of 51 patients from these three phases were included (Table 1).

**Table 1:** Clinical characteristics of CHB patients and HCs in the study

Group	IT	IA	IC	HC
No. of Cases	13	17	21	21
Gender (M/F)	10/3	11/6	17/4	8/13
Age (years)	31(27–33)	28(26–34)	36(30–39)	27(25–34)
HBsAg	+	+	+	−
HBeAg	+	+	−	−
ALT (U/L)	32.9	104	27.1	18.9
	(23.95–45.5)	(73.5–150)	(19.7–37.1)	(9.475–33.8)
HBV DNA	7.528	6.812	ND	ND
(log10 IU/ml)	(7.33–7.826)	(5.318–7.61)		

*HC* healthy controls, *IA* immune reactive HBeAg-positive phase, *IC* Inactive HBV carrier state, *IT *Immune tolerant phase, *ND* not detectable, *ALT* alanine transaminase. Data are shown as median and range

**Fig. 4:**
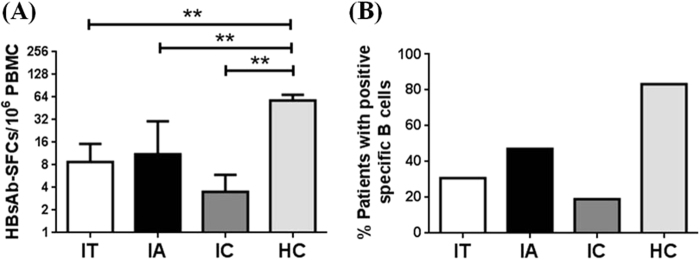
HBs-specific B cells in CHB patients. **a** The positive rates of HBs-specific B cells in immune tolerant (IT), immune reactive HBeAg-positive (IA), and inactive HBV carrier (IC) phases were expressed as a percentage. **b** The frequencies of HBsAb-SFCs among three CHB phases were displayed. **p < 0.01

Based on the B-cell ELISpot assay, the percentage of positive HBsAb-secreting B cells in IT phase of HBV patients was 30.8% (4 out of 13 IT patients), 47.1% (8 out of 17 IA patients), and 19.1% (4 out of 21 IC patients; Fig. 4a), indicating that HBsAb-secreting cells were present in all three subtypes of HBV patients but it appeared that less patients in IC phase had HBsAb-secreting B cells although the study sample size was too small to draw a conclusion. Further analysis showed that patients in IT group had an average of 8.75 ± 6.46 HBsAb-secreting B cells per 10^6^ PBMCs, patients in IA group had an average of 11.15 ± 4.67 HBsAb-secreting B cells per 10^6^ PBMCs and patients in IC group had an average of 3.5 ± 2.41 HBsAb-secreting B cells per 10^6^ PBMCs, also indicating less HBsAb-secreting B cells in IC group patients. All three groups had significantly lower frequency of HBsAb-secreting B cells than in healthy subjects (Fig. 4b).

### HBs-specific B-cell responses in HBV patients receiving one dose HBV vaccine

There were off-label uses of HBV vaccines in an attempt to induce HBsAb in certain HBV patients but no published report to confirm the clinical benefits of such practice. In the current study, we investigated whether one does of regular HBV vaccine may induce or expand HBs-specific B-cell responses in CHB patients, in addition to the traditional practice of monitoring the HBsAb responses.

Since previous literature suggested that most CHB patients undergoing HBsAb seroconversion had low level of HBsAg, and immune responses could be impaired with presence of large amount of serological HBsAg^14–16^, we hypothesized that CHB patients with undetectable HBV DNA and low level of serum HBsAg might be more capable to respond to HBV vaccination. Twelve CHB patients were recruited in this pilot study. Among them, four patients (33.33%) had serum HBsAg less than 100 IU/ml which was achieved spontaneously and treatment naive, and the other eight patients (66.67%) had received long-term anti-viral treatment and achieved good viral load suppression. All of twelve patients received one-time immunization of HBV vaccine Engerix B^®^. Their HBs-specific B-cell responses were monitored by B-cell ELISpot assay on day 0 (prior to immunization) and at day 7 and 28 after the immunization.

**Fig. 5:**
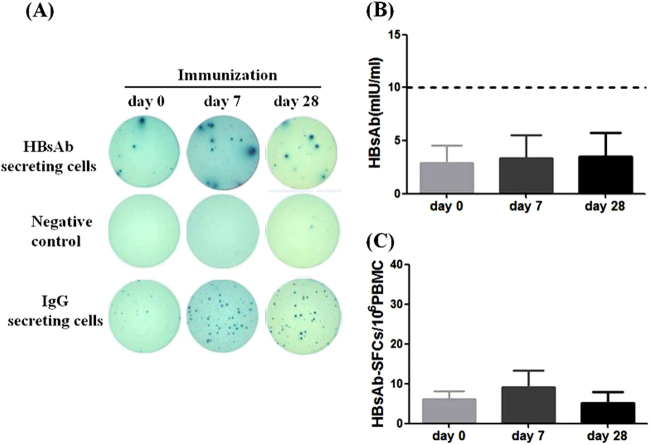
HBV Vaccine-elicited memory B-cell response in CHB patients. **a** Representative B-cell ELISpot readouts (patient VP6) at days 0 (left), 7 (middle) and 28 (right) after booster. **b** Serum HBsAb titers and (**c**) HBs-specific B cells were quantitatively measured from 12 CHB patients at days 0, 7, and 28 after receiving the HBV vaccination

Low level of HBs-specific B cells was detected in several patients even at day 0, and one-time HBV vaccine did not significantly increase the frequency of HBsAb secreting B cells as shown in patient VP6 (Fig. 5a). HBsAb testing showed below 10 mIU/mL in these CHB patients before vaccination and the HBsAb titers had little change after one dose HBV vaccination (Fig. 5b). The HBsAb-secreting B cells numbers went up first at Day 7, then dropped at day 28 after the vaccination to a level similar to day 0 (Fig. 5c). Such a pattern was different from that for healthy adults after the HBV vaccine boost (Fig. 2). Without prior history of HBV vaccination, one-time immunization in CHB patients may only prime the immune system to have HBs-specific B-cell expansion but failed to increase the production of HBsAb. The exact mechanism is not clear but the circulating HBsAg as part of active HBV infection may have some inhibitory effect. This is very different from healthy people with history of vaccination who have highly responsive HBs-specific memory B cells and can easily respond to an additional vaccine boost with higher levels of HBsAb and HBs-specific B-cell responses.

### HBs-specific B-cell responses in HBV patients receiving Pegylated interferon (PEG-IFN) treatment

**Fig. 6:**
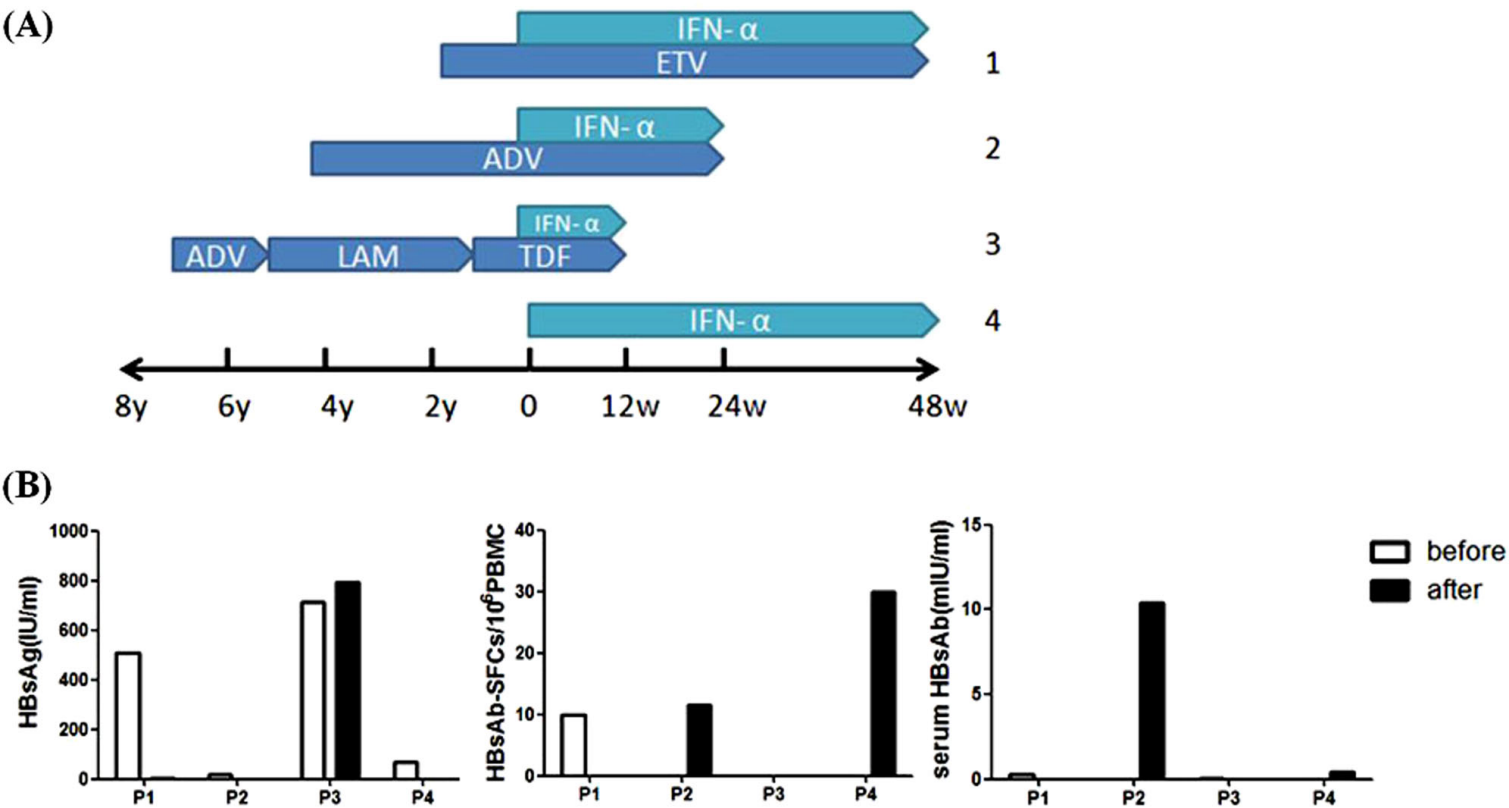
HBs-Specific memory B cell response with PEG-IFN treatment. **a** Therapeutic regimens of four CHB patients. Patient 1, 2, and 3 had combination of PEG-IFN with nucleoside analogs while patient 4 had PEG-IFN treatment alone. **b** Serum HBsAg levels, HBs-specific B cells and serum HBsAb titers before (white column) and after (black column) PEG-IFN treatment of these 4 patients were displayed. *ETV* entecavir, *ADV* adefovir dipivoxil, *LAM* lamivudine, *TDF* tenofovir

Based on the above observation, we further tested the HBs-specific B-cell responses in patients who have received PEG-IFN immune treatment and some of them had very low HBsAg in circulation before the test. PEG-IFN is a long-acting IFN which modulates body’s immune responses with the potential of inducing sustained off-treatment response and HBsAg clearance^17–19^. To examine if PEG-IFN treatment can impact the kinetics of HBsAb-secreting B cells, B cell ELISpot assay was conducted in 4 HBV patients before and after PEG-IFN treatment (Fig. 6a). Three patients (patient 1 to 3) were treated with nucleos(t)ide analogs for months to years, and then had an add-on PEG-IFN therapy for 48, 24 and 12 weeks, respectively. The fourth patient was given PEG-IFN alone for 48 weeks.

Two patients (patients 2 and 4) showed detectable HBsAb-secreting B-cell responses after either 24-week or 48-week treatment with PEG-IFN (0 vs. 11.6 or 0 vs. 30 per 10^6^ PBMCs, respectively). Patient 2 showed detectable HBs-secreting B cell responses and correlated well with the significant increase of serum HBsAb. Patient 4 showed higher detectable HBs-secreting B-cell responses after PEG-IFN treatment, but no increase of serum HBsAb at the time of test, but it is not clear whether there will be delayed HBsAb responses. On the other hand, there was no improvement on the detection of HBsAb-secreting B cells for the other two patients and none of them had detectable HBsAb responses (Fig. 6b). Interestingly, both patients who had good HBs-specific B-cell responses had very low serum HBsAg levels (Fig. 6b), further supporting the idea that circulating HBsAg may have inhibitory effect to the induction of HBsAb.

## Discussion

It is well established in literature that HBsAb can provide long-term protection in a general healthy population^20–22^. The protective role of HBsAb in clearance of circulating HBsAg and suppression of HBV replication was also reported. For example, the combination of HBV vaccine and hepatitis B immunoglobulin (HBIG) given within 12 h of birth to neonates born from HBV-infected mothers significantly diminished the rate of perinatal transmission of HBV^13^. HBV-infected patients receiving liver transplantation had a more rapid clearance of serum HBsAg, if the donor previously generated robust HBsAb responses^23,24^.

Therefore, the disappearance of HBsAg and the induction of protective HBsAb responses are considered as an optimal treatment endpoint, termed as ‘functional cure’. However, only very small percentage of CHB patients cleared HBsAg, with or without treatment^13,25^. A promising clinical observation is that, for patients who experienced the complete loss of HBsAg after decades of anti-viral treatment, they could generate decent levels of HBsAb^26^, indicating that B cells from HBV patients may still maintain the potential to produce HBsAb. In contrast, it was reported that diminished HBsAg-specific B-cell responses were correlated with HBV persistence^27^. The main challenge is how to identify those CHB patients who may be more likely than the others to develop HBsAb and clear HBsAg, and what therapeutic strategies can be developed to take advantage of such mechanism to achieve a good clinical outcome.

It was recognized that tracking HBs-specific memory B cells by ELISpot assay was more reliable than measurement of HBsAb secreted in supernatants of cultured B cells by an ELISA^22^. Indeed, since such assay was described as early as in 1983 (ref. 28), B-cell ELISpot assay has been used effectively in studying various viral infections including hepatitis C virus^29^, influenza virus^30^, human cytomegalovirus^31^, Epstein-Barr virus^32^, and dengue virus^33^. These studies provided unique information which otherwise would not be learned from the traditional antibody studies.

B-cell ELISpot method was also used to study the impact of HBV vaccination in normal people^22^ but very few such studies were done in HBV infected patients. One issue is the quality requirements for such assays to be sensitive and specific enough to measure the low level HBs-specific B cells in HBV infected people. In the current study, we followed the successful technical approach used in a recent report on HIV-specific B cells responses^34^, and adopted the same approach to the study of HBV-specific B-cell responses in both normal healthy and infected people.

In this pilot study, HBs-specific B-cell responses were measured in four clinical settings. First, we demonstrated the utility of B cell ELISpot assay to detect and quantify HBs-specific memory B cells in people previously vaccinated by HBV vaccines. The levels of B cell responses were proportional to the serum HBsAb. This finding was not unexpected but it also pointed the possibility of using HBs-specific B-cell ELISpot to monitor the immunogenicity of HBV vaccines especially for those ‘non-responders’. In this part of study, people were recruited many years after the primary vaccination course, and the effective vaccines generated protective HBs-specific B-cell memory. The ELISpot readout mainly showed the memory B cells after in vitro stimulation. It will be interesting to conduct the similar studies shortly after the end of 3-dose immunization series as well as the long-term follow-up on the dynamics of HBs-specific B cells in people who completed the HBV immunizations.

Second, the effects of one-time boosting with HBV vaccine to previously immunized healthy people were studied. The HBsAb and HBs-specific B cells with re-exposure of HBV vaccine took different responding patterns. Over the period of 28 days after the boost, the serum HBsAb went up steadily due to the continuous production of antibodies after boosting the previously immunized hosts. The HBs-specific memory B cells proliferate robustly in response to HBsAg re-exposure and promote the generation of plasma cells, which produce the circulating antibodies^35^. On the other hand, HBs-specific B cells had a prominent peak around 7 days but quickly dropped to a level still higher than the baseline level. This indicated that activated B cells differentiate into short-lived plasmablasts whose number peaks at days 6 or 7, in addition to the memory B cells. By day 28 only memory B cells became the main circulating HBs-specific B cells.

Third, we did observe very low level but clearly positive HBs-specific B cells in CHB patients at various immunological phases which were different from a previous study showing the deficiency of HBs-specific B cells in CHB patients^27^. The discrepancy might be derived from the experimental details in previous B-cell ELISpot assay. For example, *Phytolacca americana* pokeweed mitogen was used for polyclonal stimulation of PBMCs^36^, which may not be as potent as the combination of R848 and IL-2. Furthermore, the stimulation time (3 days) might not be sufficient, while in our study, PBMCs were stimulated for 5 days. Our optimized B-cell ELISpot approach used in the current study was successful in evaluating HBs-specific memory responses as that reported for HIV vaccine studies using the similar approach^34^. By using this optimized assay, we found that the levels of HBs-specific memory B cells in IA phase were much higher than in the IC phase of CHB patients. A larger sample size is needed to further confirm that the frequency of HBs-specific B cells is higher in IA than IC phases.

Finally, we studied whether immune therapies can boost the level of HBs-specific B-cell responses. When CHB patients received one dose HBV vaccine, there was basically no clear induction of HBs-antibodies. In these patients, the frequency of HBs-specific B cells was also maintained at very low level, despite a slightly higher level at day 7 with a high standard deviation. Therefore, receiving one-dose HBV vaccine in CHB patients is not likely to bring desired immunological responses. Future studies can be conducted with additional doses of vaccine and the B-cell ELISpot assay can be used to monitor the immune responses.

In our study, we also provide the first evidence that PEG-IFN, a non-antigen-specific immune treatment induced HBs-specific memory B-cell responses in at least two patients. Potent antiviral drugs could efficiently suppress viral replication via inhibiting the reverse transcriptase or HBV polymerase; while PEG-IFN modulated the immune-mediated control of HBV infection, providing an opportunity to achieve HBsAg clearance. Previous studies reported that 30% of patients treated with PEG-IFN for 48 weeks experienced HBeAg seroconversion, whereas only 3–7% fully cleared HBsAg in other clinical trials^37–39^. In one hospital-based study in Asia, PEG-IFN was added after 24 weeks of entecavir therapy and improved the response rate, as demonstrated by HBeAg loss and declines in HBV DNA^40,41^. Further, high rates of HBsAg clearance and seroconversion could be achieved by PEG-IFN-based treatments even in inactive HBsAg carriers among whom treatment was not traditionally recommended^17^. In current study, we demonstrated that PEG-IFN treatment was able to expand the HBs-specific B cells in CHB patients. Future studies with much large study cohorts should recruit CHB patients with suppressed HBsAg who will then receive PEG-IFN treatment and the development of HBs-specific B-cell responses will be monitored in addition to the study of HBsAb. In our current study, one patient developed HBs-specific B cell response but no obvious HBsAb responses which may indicate the later development of antibodies than the B-cell responses. If it is true, then the HBs-specific B-cell responses can be used as an early indicator of good therapeutic responses.

In summary, the current report described the useful applications of a sensitive and specific B-cell ELISpot assay to study the immune responses in healthy and HBV-infected patients. Findings in this pilot study also pointed to several potential directions to test the effects of antigen-specific and non-antigen-specific immune therapy strategies when the HBs-specific B-cell ELISpot is used to follow the clinical outcome of such immune interventions. At least, ELISpot assay can be further improved by including phenotypic cell markers thus it can provide additional information to understand the immunological mechanisms that are involved in the induction and maintenance of HBsAb in the various clinical settings.

## Materials and methods

### Study participants

The healthy control group (HC) included 21 adult volunteers who completed the standard HBV vaccine immunization (three doses) at their childhood. Seven of them received one-time boost of HBV vaccine Engerix B in the current study.

A total of 67 HBV-infected patients were enrolled in this study. Patients with end-stage liver insufficiency, autoimmune disorders, or malignancies were excluded from the study. Patients who were positive for antibodies against other types of hepatitis or human immunodeficiency virus were also excluded. These patients were included in several studies. In one study, 51 HBV patients who did not receive any antiviral treatment (‘treatment naïve’) were further divided into three subgroups: 13 in immune tolerant (IT) subgroup, 17 in immune reactive HBeAg-positive (IA) subgroup, and 21 in inactive HBV carrier (IC) subgroup based on the 2012 clinical guidelines prepared by the European Association For The Study Of The Liver (EASL)^13^. In another study, 12 CHB patients who had relatively low HBsAg level (<100 IU/mL) received one-time HBV vaccine Engerix B immunization. The final study included four chronic HBV patients who received either PEG-IFN treatment alone (one patient) or in combination with nucleoside analog drugs (three patients).

All participants were provided with a written informed consent for blood sampling and HBV vaccination under a human clinical study protocol approved by Medical Ethics Committee of Nanjing Drum Tower Hospital.

### PBMC isolation

PBMCs were prepared from 10 mL fresh heparinized study subjects’ peripheral blood specimens by density gradient centrifugation using a Ficoll cushion. Briefly, blood diluted with phosphate-buffered saline (PBS) was added into Ficoll and 10 × 10^6^ PBMCs were isolated after centrifugation. Aliquots of PBMCs were stored using Cryostar Freezing Kit (Cat#: UH-M1002-050, DAKEWE, Beijing, China) in liquid nitrogen until being used for B-cell ELISpot assay.

### PBMC culture and B-cell stimulation

After thawing, PBMCs were resuspended in RPMI 1640 medium (Gibco, Invitrogen, Paisley, UK) at 2 × 10^6^ PBMCs/mL supplemented with streptomycin, glutamine, and 10% fetal bovine serum (FBS). To effectively stimulate B cells, 1 μg/mL R848 and 10 ng/mL recombinant human (rh) IL-2 (Mabtech, Nacka Strand, Sweden) were added. Cells were subsequently incubated for five days at 37 °C, 5% CO_2_, followed by washing with RPMI 1640 medium prior to plating.

### B-cell ELISpot assay

Sterile 96-well Multiscreen-IP filter plates with a PVDF membrane (Millipore Corp, Bedford, MA, USA) were coated with either anti-human IgG (15 μg/mL, Mabtech, Nacka Strand, Sweden) or recombinant HBsAg (10 μg/mL) overnight at 4℃. Numbers of total IgG + secreting cells were determined by wells coated with capture anti-human IgG, while numbers of HBsAb secreting B cells were examined by wells coated with HBsAg. Wells with no coating were used as negative controls. Plates were washed with sterile PBS and blocked with RPMI-1640 medium/10%FBS for 2 h at room temperature. Serially diluted pre-activated cells were added at 5000 or 2500 cells/well for IgG coated wells, 100,000 cells/well for no coat wells, and 100,000, 200,000, or, 400,000 cells/well for HBsAg-coated wells. The culture plates were incubated for 18 h at 37 °C, 5% CO_2_. Each condition was performed in duplicates. Frequency of antibody-secreting cells was measured after washing with PBS and incubation with biotin-labeled anti-IgG mAb (Mabtech, cat#3850-2 H, Sweden) and horseradish peroxidase (HRP)-conjugated streptavidin (Mabtech, Nacka Strand, Sweden). An automated ELISpot image analyzer (Cellular Technology Limited, Hongkong, China) was used to analyze and count the spots. Frequency of HBsAb-secreting B cells per well was calculated by subtracting background values from negative control wells.

### PEG-IFN treatment

Managed as part of the regular clinical treatment by infectious disease specialists, 4 HBV patients were treated with PEG-IFNα-2a (Pegasys, Roche, USA). Three of them received the add-on PEG-IFN after months or years of treatment with nucleos(t)ide analogs, while the 4th patient did not have previous history of HBV treatment and received PEG-IFN treatment alone during the course of current study. The blood samples were collected before and after the PEG-IFN treatment.

### HBV vaccine administration

Certain groups of subjects received one-time intramuscular injection of HBV vaccine Engerix B® (20 µg HBsAg protein, GlaxoSmithKline, Brentford, UK) during the study period. Heparinized blood (10 ml) was obtained on day 0 (prior to immunization), at day 7 and day 28 post immunization for serum antibody and B-cell ELISpot analyses.

### Assessments of HBV serologic biomarkers

Routine HBV serologic markers (HBsAg, HBsAb, HBeAg, HBeAb, and HBcAb) were determined using Architect-i2000 system (Abbott Laboratories, USA). The quantitative determinations of biomarkers were considered positive according to the criteria set by the manufacturer.

### Statistical analysis

All the above experiments were done in triplicate. Final data were analyzed statistically if indicated by using the SPSS 22.0 software. Most data were presented in form of mean ± s.e.m. Results with *p*-value <0.05 were considered to be statistically significant.
